# Automatically clustering large-scale miRNA sequences: methods and experiments

**DOI:** 10.1186/1471-2164-13-S8-S15

**Published:** 2012-12-17

**Authors:** Linxia Wan, Jiandong Ding, Ting Jin, Jihong Guan, Shuigeng Zhou

**Affiliations:** 1 School of Computer Science, Fudan University, Shanghai 200433, China; 2 Department of Computer Science and Technology, Tongji Uinversity, Shanghai 201804, China; 3 Shanghai Key Lab of Intelligent Information Processing, Fudan University, Shanghai 200433, China

## Abstract

**Background:**

Since the initial annotation of microRNAs (miRNAs) in 2001, many studies have sought to identify additional miRNAs experimentally or computationally in various species. MiRNAs act with the Argonaut family of proteins to regulate target messenger RNAs (mRNAs) post-transcriptionally. Currently, researches mainly focus on single miRNA function study. Considering that members in the same miRNA family might participate in the same pathway or regulate the same target(s) and thus share similar biological functions, people can explore useful knowledge from high quality miRNA family architecture.

**Results:**

In this article, we developed an unsupervised clustering-based method miRCluster to automatically group miRNAs. In order to evaluate this method, several data sets were constructed from the online database miRBase. Results showed that miRCluster can efficiently arrange miRNAs (e.g identify 354 families in miRBase16 with an accuracy of 92.08%, and can recognize 9 of all 10 newly-added families in miRBase 17). By far, ~30% mature miRNAs registered in miRBase are unclassified. With miRCluster, over 85% unclassified miRNAs can be assigned to certain families, while ~44% of these miRNAs distributed in ~300novel families.

**Conclusions:**

In short, miRCluster is an automatic and efficient miRNA family identification method, which does not require any prior knowledge. It can be helpful in real use, especially when exploring functions of novel miRNAs. All relevant materials could be freely accessed online (http://admis.fudan.edu.cn/projects/miRCluster).

## Background

Over the last decade, 20-30 nt RNA molecules have emerged as critical regulators in the expression and function of eukaryotic genomes [1,2]. microRNA (miRNA), one of the most important categories of these small RNAs, acts in both somatic and germline lineages in a broad range of eukaryotic species to regulate endogenous genes and to defend the genome from invasive nucleic acids [3-6]. In 1993, the first identification of *lin-4 *in *C. elegant *added another dimension to the known genome regulation networks [7]. miRNAs act as guide molecules in post-transcriptional gene silencing (PTGS) by base pairing with target mRNAs, which leads to mRNA cleavage or translational repression [8].

The biogenesis of both animal and plant miRNAs is a two-step process [9,10], which initiates with the nascent transcripts - primary miRNAs (pri-miRNAs) that are usually several hundred nucleotides long. For animal miRNAs, the primary transcripts are processed in the nucleus by a multiprotein complex containing an enzyme called *Drosha *to give rise to the ~70 nt long miRNA stem-loop precursors (pre-miRNAs) which are then exported to the cytoplasm [10]. In the cytoplasm, a second step takes place where a pre-miRNA matures into a ~21-nt long miRNA:miRNA duplex, with each strand originating from opposite arms of the stem-loop. Although some miRNAs are Dicer independent [11,12], most are produced by the action of an enzyme called Dicer, which recognizes the double-stranded stem [13]. In general, the miRNA strand is then integrated into the miRNA-induced silencing complex (miRISC) or miRNA-containing ribonucleoprotein particles (miRNPs) and the miRNA* is degraded [14]. Sometimes both strands can be detected, in which case the miRNA* designates the less predominant form of the mature miRNA [15,16].

In plants, miRNA biogenesis follows a similar process, but a miRNA seems to be fully matured into a single stranded miRNA before being exported to the cytoplasm and integrated onto the silencing complex, which partially explains why intermediate forms of plant miRNAs are only rarely detected [9,17]. All maturation steps of plant miRNAs are processed by Dicer-like proteins [18]. Plant miRNA precursor sequences are much more diverse in both length and secondary structure than those in animals [19]; there are two precursor-processing pathways that have been identified for plant miRNA genes. Besides the primary pathway involves stem-to-loop processing [20,21], the second pathway involves loop-to-base processing in which the sequence and structure beyond the miRNA-miRNA* site are necessary and used by the cleavage pathway components to excise the mature sequence(s) [22,23].

**Figure 1:**
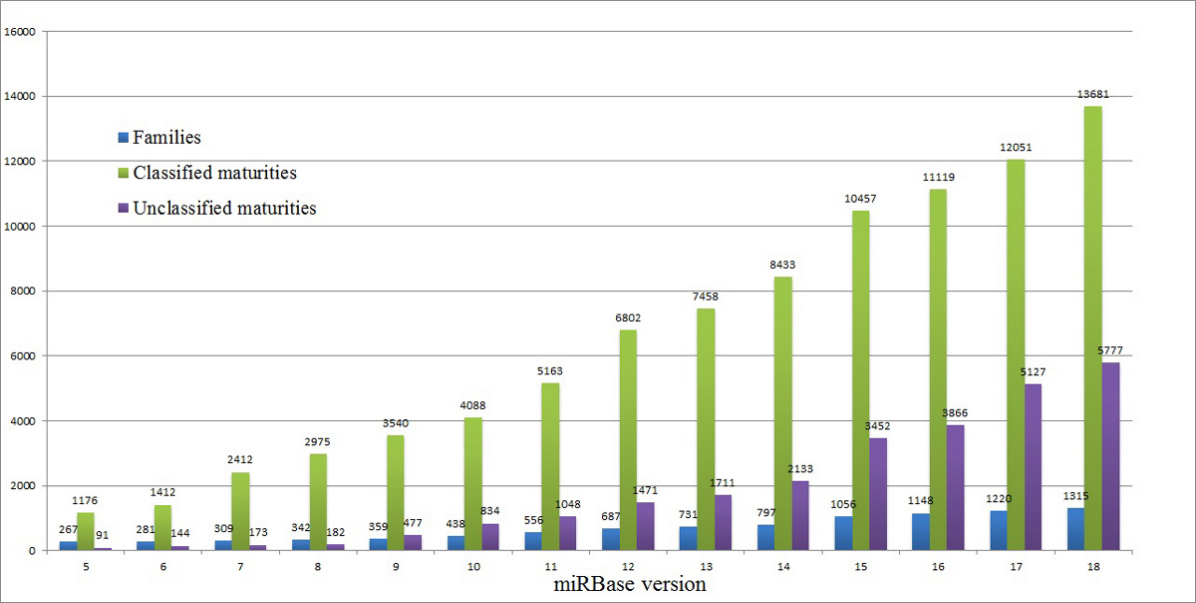
**Rapid growth of miRNA genes**. The number of miRNAs registered in miRBase increased rapidly these days. We explored the unclassified and classified mature miRNAs along the development of miRBase from version 5 to 18. The numbers of families were also listed.

After the initial wave of miRNA identification, a small portion miRNAs were estimated to be encoded in various genomes [24-26], but it was subsequently recognized that this estimate could be low [14]. Later studies, based on combinations of computational and experimental techniques, support a substantially larger number of miRNAs [27-29]. Advances in technology and methodology, especially the appearance of Next Generation Sequencing (NGS) technologies [30] leads to the description of thousands of candidate miRNA genes. Several methods and many pipelines are proposed to analyse sequences from small RNA deep-sequencing data sets to see if they meet a given set of rules [31,32]. If a sequence meets the necessary rules and the surrounding sequence is able to fold into a stem-loop like structure, then it is automatically classified as a new miRNA [33-35]. As the most authoritative online database, miRBase collects and registers all experimentally validated miRNAs and computationally predicted candidates. The number of microRNAs deposited in miRBase has been increasing approximately exponentially. In the last 3 years alone, the number of microRNA sequences in the database has almost trebled [36,37]. At the time of writing this paper, miRBase (release 17) contains over 16,700 microRNA loci, expressing over 19,000 distinct mature sequences, from 170 species. From the 5^th ^version, miRBase began to provide miRNA family information, which means to gather miRNAs sharing similar primary sequence and/or secondary structure into one group. Current semi-automated procedures for miRBase to build miRNA families from submitted data and supplementary data of publications have not been sufficient to keep pace with the increasing rate of miRNA identification (Figure 1). Recently, Ding *et.al*. proposed an effective alignment free model named *miRFam *to classify miRNAs based on the combination of *n*-gram and multiclass SVM [37]. As the first miRNA-oriented family classification method, it extracts *n*-grams as features from primary sequences. When measuring sequence similarity, it uses shorter sequence segments, which allows it to run faster. Results show that the classification method can always achieve acceptable performance (e.g. it can arrange 9,379 pre-miRNAs to 398 families with an accuracy of 97.97%). Currently, there are still two remaining limitations for this classification-based method. On one hand, it relies on the existing family architecture. On the other hand, novel miRNAs that do not belong to any existing family will be misclassified. With the hope to overcome these limitations, we develop miRCluster, a powerful unsupervised clustering-based method. Although it also uses *n*-grams to describe miRNA sequences, comparing with the former method, miRCluster has several advantages: 1) No prior family architecture information is required. miRCluster can directly assign one miRNA to its corresponding family automatically based on its primary sequence. 2) By considering larger *n*-grams, miRCluster is developed to treat much shorter mature sequences. 3) The most appropriate family number is determined dynamically, which make miRCluster accurate and effective. 4) Advanced technologies are employed to choose more representative features, which thus dramatically improves the processing speed and also slightly improves the performance.

## Methods

### Datasets

In this paper, we intend to develop a method to automatically arrange miRNA maturities, especially after noticing that not all miRNAs in miRBase have been classified by far. For example, from version 5 to 18, the number of classified miRNAs increases from 1176 to 13,681, while unclassified matures increases much faster from 91 to 5,777 (see Figure 1).

We first analysis miRNA families in miRBase16 and miRBase17 (see Table 1). There are 1,148 families and 14,985 miRNAs in miRBase16, while the two numbers are 1,220 and 17,178 in miRBase17. Currently, those families can be further divided into 4 biological organisms: animal, plant, viruses and chromalveolata. But the latter two organisms are so small since they only contain 33 and 1 families respectively in miRBase16, and most families contain less than 5 members. In this study, to get a convincing and comprehensive view, only animal and plant families that contain no less than 4 members are selected to be further studied.

**Table 1:** Statistical results of miRNA families in miRBase16 and miRBase17

miRBase		miRBase16	miRBase17
Species		142	153

Family numbers		1148	1220

Family number of each organism	animal	858(319)*	914(331)
	plant	256(75)	268(82)
	viruses	33(2)	37(2)
	chromalveolata	1(1)	1(1)

* For example, 858 (319) means that there are 858 animal families, and 319 of them contain no less than 5 members.

According to the different motivations of our study, including feature selection test on miRBase16, new family evaluation on miRBase17 and novel family discovery from unclassified miRNAs on miRBase17, we construct three datasets based on miRBase16 and miRBase17. In order to simplify the description, we used some notations to represent them (see Table 2). First, in order to test whether feature selection could improve the performance of miRCluster, we construct R1 by selecting 394 animal and plant families (at least 5 members) that contain 82.97% (9,225/11,119) of all classified mature sequences in miRBase16. Second, in order to assess its ability to discovery new families and new members we construct dataset R2 by adding 605 new members of existing families and 10 new families (at least 4 members) that are added in miRBase17 to R1. Finally, R3 was built by miRNAs of 413 existing animal and plant families and all 5,127 unclassified mature sequences in miRBase17.

**Table 2:** Notations of datasets

	Notation	Description
Feature selection on miRBase16	R1	Animal and plant families that hold at least 5 members in miRBase16
New families evaluation in miRBase17	R2	Besides R1, also includes new members of novel families and existing families (contain no less than 4 members) in miRBase17.
Prediction of unclassified members in miRBase17	R3	Animal and plant families that contain no less than 5 members, plus unclassified matures in miRBase17.

### Feature extraction and feature selection

In this paper, we develop an effective clustering-based method to automatically build up a family system of miRNA maturities, and compare the result with the family system provide by miRBase. Here, *n*-grams [38] is used as the feature extraction method to transform primary sequences to numeric vectors. We then consider several feature selection methods to reduce the dimension of feature matrix, thus get a smaller group of more informative features.

#### Feature extraction

An *n*-gram is a subsequence consisting of *n *spatially consecutive items extracting from a given sequence [38]. Here, the items are base nucleotides A, C, G and U. Considering the instinct difference between these features, we adopted a weighted concentration method from miRFam [37] to combine these features into one vector, , and all values sum up to 1 in every single feature vector. To facilitate the illustration, we used the notation of "GramN" as the combination of 1-gram, 2-gram...and N-gram features. Consequently, Gram4 or Gram5 are chosen as features in different datasets for our study.

#### Feature selection

As explained above, there are 4 features of 1-gram, 16 of 2-gram, 64 of 3-gram, 256 of 4-gram, and 1024 of 5-gram respectively. However, the disadvantage might be that: some redundant features or noise data may exist among the large feature space. In order to effectively select a subset of representative features and construct a more robust family discovery method, three different dimension reduction methods are introduced here. Detailed information about them is as follows.

Latent semantic analysis (LSA) [39], a famous method in natural language processing, which extracts and represents the contextual usage meaning of words by statistical computations applied to a large corpus of text. At the beginning, a matrix of word counts per document is constructed. Then, the particular technique singular value decomposition (SVD) is performed to reduce the columns while preserving the similarity between rows. LSA has been greatly successful in many information retrieval applications such as document classification [40], spam filtering [41], and text summarization [42].

Locally linear embedding (LLE) [43] is a recently proposed unsupervised learning algorithm to compute low dimensional embedding of high dimensional data, while neighbourhood relationship is preserved. It computes the *k *nearest neighbours of each data point in *D*-dimension at first, and then generates a weight matrix that best reconstructs each data point from its neighbours, and finally computes the *d *dimensional embedding coordinates that reconstructed by the weight matrix.

Isometric feature mapping (Isomap) [44], another low-dimensional embedding method, is widely used by incorporating geodesic distances on a weighted graph with metric multidimensional scaling. It consists of three main steps. First, it constructs a neighbourhood graph after computing *k *nearest neighbours, and then geodesic distances are estimated between all pairs of points by computing the shortest paths in the graph. Finally, an embedding is constructed in *d *dimensions that best preserves the estimated geometry in the second step.

There are some differences among them. LSA is a method that basically uses SVD to reduce the dimension according to singular values, while LLE and Isomap both compute low dimensional embedding based on *k *nearest neighbours. LLE is aimed to maintain the local linear reconstruction relationship among points; while on the other hand, the major concern of Isomap is to get a low-dimensional representation that best preserves geometry computed from the graph.

Based on their performances on dataset R1, the one with best performance is chosen to be used in the following experiments.

### Clustering method and evaluation

The main aim of this paper is to develop an effective method to automatically discover miRNA families by performing unsupervised clustering analysis. In this study, we adopt a simple *K*-means [45] clustering method from Biopython [46], which is a set of freely available tools for biological computation written in python. Currently, there are many distance measurements available to evaluate the relationship between two data points [47], such as Euclidean distance, city block distance, and spearman's distance. However, after some tests, "city block distance" is chosen here as it always gives better clustering results. To evaluate the performance of our clustering approach, we provide two measure metrics. Generally, the most straightforward way to evaluate the performance of a clustering method is F-measure [48]. At the beginning, F-measure is computed to balance both precision and recall values for each family. Then, an average F-measure can be calculated by weighting different family size to quantify the agreement between true families and generated clusters. Besides, we also consider a specific accuracy score after adopting a "Vote strategy" to further explore those generated clusters. It works as follows. First, small clusters with less than 5 miRNAs are thought to be of bad quality and ignored. Second, after calculating detailed distribution of families in each cluster, we assign a cluster to the family that has the largest number, and only those miRNAs are thought as correctly clustered. However, in some cases, several miRNAs from different families (e.g. plant families MIR169_1 and MIR169_2) are so similar that they are clustered together frequently. Hence, in each cluster, if the number of the second largest family exceeds 1/3 of the largest one, we will also treat that meaningful family as correctly clustered.

Unfortunately, there're still two limitations of *K*-means algorithm. On one hand, it is significantly sensitive to the initial randomly chosen cluster centres. To reduce this unstable factor, we run the clustering procedure multiple times (e.g. 20 here) in each experiment, and eventually choose the one with best convergence. On the other hand, it is difficult to determine the number of clusters in a specific dataset without any prior information. For most of clustering methods, this parameter needs to be determined by users. To solve this problem, we perform the clustering process several times with different cluster number. Concretely, for a specific dataset if the family number defined by miRBase is *N*, the argument of cluster number *K *is set to 6 values that uniformly distributed between *N *and 2*N. For example, if there are 300 families in a dataset, then the testing cluster number is set to 300, 360, 420, 480, 540, and 600, consecutively. At last, the one with the highest accuracy score is chosen as the overall accuracy of *K*-means clustering approach.

### MSA implementation

We also use Clustal X2 (Version 2) [49] to do multiple sequence alignment of some miRNA samples during evaluating the discovering novel families from unclassified miRNAs by in miRBase17 our miRCluster.

## Results

**Figure 2:**
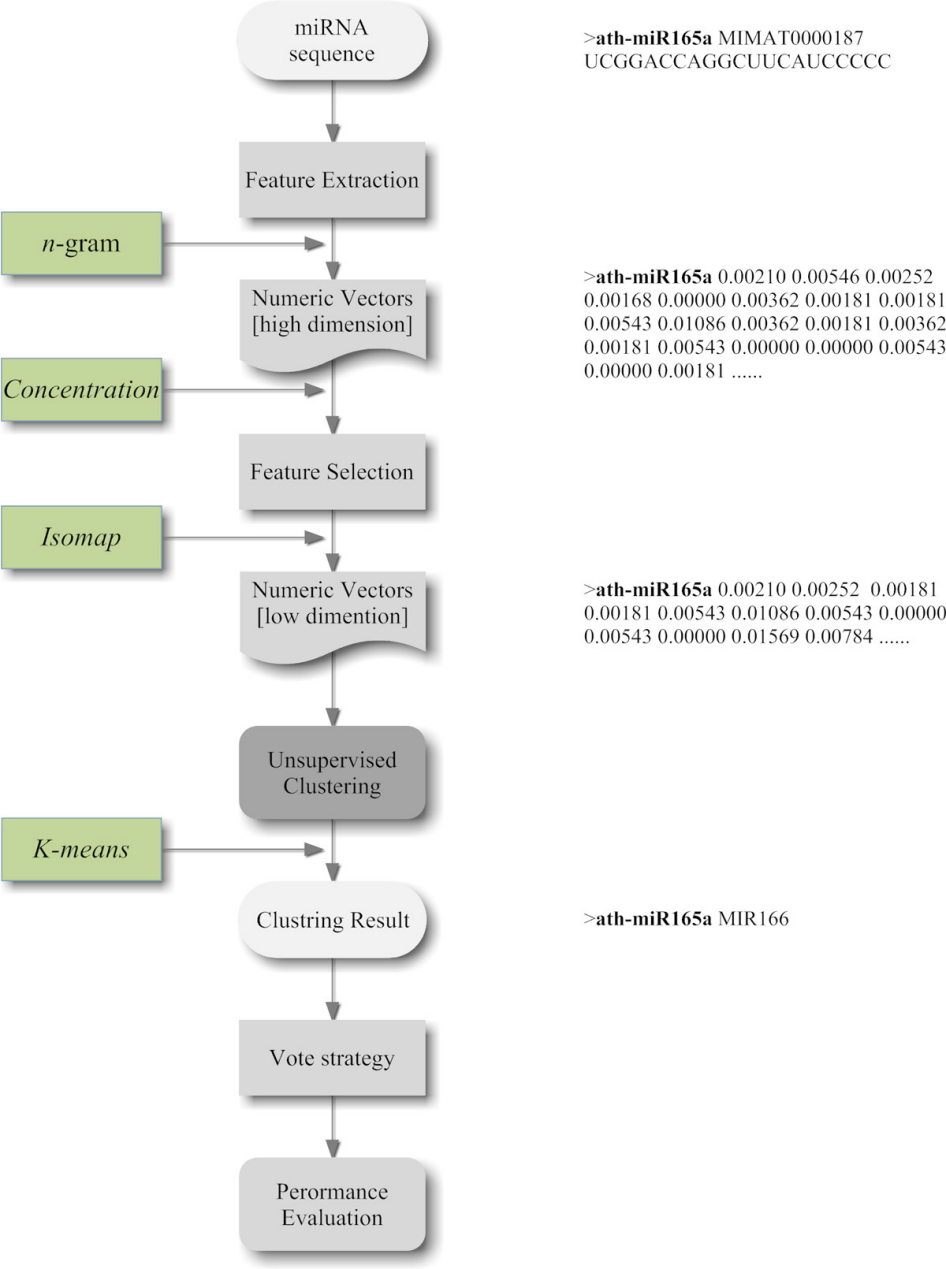
**The flow char of miRCluster**. To effectively establish a family system of miRNA sequences, we designed a pipeline miRCluster. This pipeline could be mainly divided into four steps, including feature extraction, feature selection, ***k***-means clustering and vote strategy.

In this work, we design a pipeline (show in Figure 2) miRCluster to identify miRNA families, which mainly consists of 4 stages: feature extraction, feature selection, *K*-means clustering and vote strategy. Experiments are arranged into three groups.

We start with the feature selection test. The methods used here are latent semantic analysis (LSA), locally linear embedding (LLE) and Isometric feature mapping (Isomap). With selected features falling between 10 and 180, experimental results showed that Isomap achieves the best clustering accuracy on our datasets. Therefore, we chose Isomap as the feature selection method for all the following study. The second experiment is to evaluate miRCluster's ability to identify new families and new members of existing families in miRBase17. Result shows that miRCluster can correctly detect novel families and new members. From this point of view, it is a good candidate to help annotating miRNAs when enormous new miRNAs are registered in miRBase these days. The purpose of the last experiment is to discover novel families from those unclassified miRNAs. Currently, there are 5,127 unclassified miRNAs in miRBase17, which account for almost 1/3 of all the mature sequences, thus it is a great challenge to find meaningful results by mining these unclassified miRNAs.

### Feature selection on miRBase16

In miRBase16, there are about 1,114 animal and plant families, but more than half are too small that contain less than 5 members. As we all know, for most clustering methods, it is hard to perfectly separate small families from big families which contain the majority of miRBase. Hence, small families are ignored, and finally we get 319 animal and 75 plant families, of which the total mature sequences are 7,197 and 2,028, respectively. For example, the three biggest families in animal are let-7, mir-17 and mir-154, which hold 195, 175 and 169 members respectively. Meanwhile, the top 3 families in plant are MIR166, MIR156 and MIR395, which have 141, 140, and 119 miRNAs respectively.

In our analysis, Gram4 (combination of 1, 2, 3, 4-gram) is extracted, followed by using a concentration method illustrated in *miRFam *[37] to get original features. With *K*-means clustering and vote strategy, we can calculate two performance metric values: F-measure and accuracy. The average F-measure is 0.7737, and accuracy is 91%, which means 8,395 mature sequences are correctly clustered and 830 are not.

Since the original feature vector contains 340 high dimensions, direct clustering approach is always time-consuming and accuracy-lacking. Hence, we consider three dimension reduction methods: LSA, LLE and Isomap. The parameter of *k *nearest neighbours for LLE and Isomap is set to 10, and the selected dimensions are tested from 10 to 180 with a step size 10.

**Figure 3:**
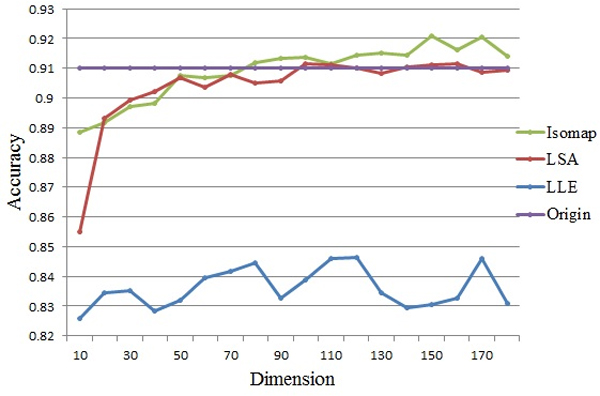
**Accuracy result of feature selection (achieved on miRBase16)**. Here, we treat the feature selection as a matrix dimension reduction issue and considered three different methods: LLE, LSA and Isomap. The horizontal axis is the reduced dimension, and the vertical axis is the clustering accuracy. The "Origin" line stands for the performance of clustering result with all ***n***-gram features, while others are results of accuracy after feature selection with different methods.

**Figure 4:**
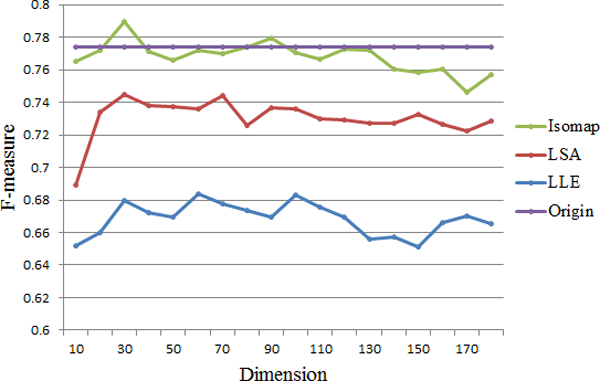
**F-measure result of feature selection (achieved on miRBase16)**. Again, the horizontal axis is the reduced dimension, while the vertical axis is the F-measure value. Here, only results of three feature selection methods are shown. Consistent with the accuracy result, Isomap achieves the best result.

Figure 3 shows the accuracy before and after feature selection using the three methods. We find that the clustering approach performs even worse than before when LLE is used, as the accuracy is always less than 85%. Hence, LLE is not suitable here, although better performance might be achieved by carefully adjusting the parameters. As for LSA, its accuracy is even lower than before when dimensionality is under 90, and doesn't improve much with dimensionality increases. Overall, we find that miRCluster performs better after using Isomap, and achieves a highest accuracy of 92.08% when 150 features are selected. And over-fitting problem is unlikely to happen since Isomap does not rely too much on the specific value of each dimension from the original data. Apparently, the F-measure result in Figure 4 also demonstrates that Isomap outperforms the other two methods.

According to the above experimental results, Isomap is employed for feature selection in our clustering approach, and the dimensionality could be set between 120 and 160, in order to get the balance of time and accuracy.

Additionally, we calculate the "dead" families, which are not discovered by miRCluster due to two reasons. One is the filtering of noisy clusters (contain less than 5 members), and the other is the difficulty of distinguishing small families from bigger ones. Results are summarized in Additional file 1 Table S1. Before feature selection, we find 42 "dead" families containing 285 miRNA sequences. However, after using Isomap to select 150 informative features, "dead" families are reduced to 40, which contained 270 mature sequences. For example, mir-1422 (containing 18 members) is successfully discovered after using Isomap.

In summary, the result shows that Isomap is effective to improve the overall accuracy of our clustering approach, and it succeeds in finding some families that are not discovered before. Furthermore, experiments in later sections will demonstrate its effectiveness again.

### Evaluate new families on miRBase17

In this section, we try to evaluate miRCluster's capability to find new families and new miRNAs that belong to existing families.

There are 72 new families and 2,383 new miRNAs added to miRBase17 compared to miRBase16. Among the new families, only 10 hold at least 4 members and the total number of mature sequences is 58. It consists of a 12-member family (mir-3851), a 10-member family (mir-3811), an 8-member family (MIR5067), and 7 4-member families (Additional file 1 Table S2). Besides, there are 605 new mature sequences which belong to existing families (holds at least 5 members). By adding these data to R1, a new dataset R2 is constructed and used to evaluate miRCluster.

Again, we validate our clustering approach on the original and Isomap feature selection dataset (see Table 3). Since some new families have only 4 members, here the minimum number of members that form a meaningful cluster is set to 4.

**Table 3:** Result of evaluating new families in miRBase17

Result	Correctly clustered families	Correctly clustered members	Correctly clustered new miRNAs belonged to existing families
Before feature selection	9	34	447
After feature selection	8	37	435

After selecting 140 features with Isomap, the number of correctly clustered miRNAs that belonged to these new families increases from 34 to 37, while the number of correctly clustered new families decreases from 9 to 8.

For the dataset with all features, miRCluster find 9 new families that contain 34 new mature sequences. After selecting features with Isomap, we find that the clustering approach successfully found 8 new families containing 37 mature sequences when 140 features are selected..

Although the number of correctly found new families decreases from 9 to 8 after feature selection, more members of the two big families (mir-3581 and mir-3811) are identified. In fact, all the members in mir-3811 have been identified, while only half are discovered before. On the other hand, two small families (mir-3836 and mir-3817) each with 4 members disappear after feature selection.

Meanwhile, miRCluster identify 447 new members of existing families and this number does not change a lot before and after feature selection. All the results indicate that our method can correctly discover new families and new miRNAs. Concerning the fast growing of new miRNAs these days, miRCluster will be helpful in real use.

### Investigation on unclassified miRNAs in miRBase17

After analysing miRBase carefully, we find that many miRNAs are not classified to any known family, and the number of unclassified miRNAs increases even faster than that of classified miRNAs. For example, in miRBase 5.0, the ratio of unclassified mature sequences over classified is 7.7% (91/1,176). However, the ratio increases to nearly 42% (5,127/12,051) in miRBase17. Similar to the above experiments, we first select the 331 animal and 82 plant families that hold no less than 5 members from miRBase17, and all then unclassified miRNAs to build dataset R3.

Here, we mainly focus on 3 types of clusters. First, those cluster called "specific novel family" whose members are mainly novel miRNAs. Second, the cluster mixed by novel and existing family members at a considerable rate: member in the second largest family is more than 1/3 of member in the largest family. Lastly, the cluster constructed by novel miRNAs and an existing family, but these novel miRNAs not enough to form quality family.

All the results are shown in Table 4. First, with Gram4, miRCluster discover 231 novel families (contain 1,820 sequences), and 320 mixed families (contain 2,141 sequences) when the cluster number is set to 800. By increasing cluster number to 1,200, the discovered novel families rise to 293 (contain 1,828 sequences), but mixed families decrease to 263 (contain 1,457 sequences). Hence, we guess that novel miRNAs may be easier to be distinguished from existing families when a larger number of clusters are considered.

**Table 4:** Evaluating unclassified miRNA in miRBase17

Features	Cluster number	To novel family^1^	To mixed family^2^	To existing family	Failed
Gram4	800	231/1820	2141/320/3091	975	191
Gram4	1200	293/1828	1457/263/2299	981	861
Gram4(140 features, Isomap)	1200	301/2266	1272/205/1776	859	730
Gram5(140 features, Isomap)	1200	316/2299	1085/179/1483	935	808

^1^x/y stands for x families contain y members.

^2^x/y/z stands for x novel miRNAs mixed with z members that come from y existing families.

Below, cluster number is set to 1,200 all the time. With Isomap, we reduce the dimension to 140, this time the number of novel families increases to from 293 to 301. However, about 400 more miRNAs are clustered into these novel families, and the number of novel miRNAs mixed with known families decreases to 1,272 (1,457 before feature selection). This indicates that feature selection is a good choice to select informative features that make novel miRNAs easier to be clustered.

In all previous experiments, Gram4 is chosen. However, the relative position information is ignored because *n*-gram does not consider the specific position of a small fragment in a sequence. After exploring the clustering results by ClustalX, we find that some dissimilar sequences with only several similar fragments at different positions are frequently clustered together. Hence, we use Gram5 (1,346 features) to examine how clustering performance will be impacted by longer features. With 140 features selected by Isomap, we find that the number of novel miRNAs mixed with known families decreases by 187 compared to the result with Gram4. It seems that miRCluster performs better when longer *n*-grams are considered.

In order to clearly show this problem, we use ClustalX to do multiple sequence alignment on some clusters.

First, an example of novel family is explored (Additional file 1 Figure S1). We add a "*" in front of the sequence name to indicate it is a novel miRNA. This figure clearly shows that miRNAs in the novel family are similar to each other, and this novel family is clustered with good quality.

Then, we look a cluster that is mixed by novel miRNAs and members of known family (Additional file 1 Figure S2). This family was not discovered when the cluster number was set to 800 with Gram4, but was well established in other 3 experiments. By considering larger *n*-grams and bigger cluster number, more novel miRNAs (mmu-miR-3966, sme-miR-745, cte-miR-745a, bfl-miR-22, and pma-miR-22b) were grouped to the known family miR-22.

## Discussion

As the final product of the complicated biogenesis, biologically speaking, mature miRNAs are more important than their precursors. Two reasons make it a more challenge problem to classify mature miRNAs. First, sequence length of maturity is usually only 1/3 to 1/20 of its precursor. Second, one single pre-miRNA can generate several distinct mature miRNAs that belong to different families. By considering larger *n*-grams, miRCluster can achieve acceptable accuracy (always over 90%), but the side effect is also obvious. In order to improve the efficiency of our method, we do not directly use traditional machine learning methods to select featured *n*-grams. Instead, we treat this as a dimension reduction problem to reduce the search space. After testing three different reduction algorithms, results show that both LSA and Isomap can get comparable results while compared with the original situation when only less than 10% *n*-gram features are selected. Compared with *miRFam*, miRCluster employs similar number of features but it does not rely on any prior knowledge.

As the core functional region of miRNAs, previous studies have revealed that seed region is more conserved than other backbone regions [14,18,50]. We also constructed a weighted feature vector that emphases the seed region by calculating twice those *n*-grams extracted from this locus. Surprisingly, experiment results do not always turn better, which reveals that the seed region might not be so conserved, especially for animal miRNAs (see Additional file 1 Table S3).

Currently, around 30 percentage (5,127/16,772) miRNAs registered in miRBase (version 17) are unclassified. And the portion is becoming larger (Figure 1). In the foreseeable future, along with the development of advanced sequencing technologies and development of relevant analysis technologies, more novel and specific miRNAs will be identified from more re-sequenced or de novel sequenced species. How to manage and arrange those data automatically and effectively will be greatly helpful for miRNA research community. By far, the main part of current miRNAs family architecture is constructed by small families whose member is less than 5. And we all know that this issue is determined by the diversification of MIRNA genes and their functions. With the proposed miRCluster, we found that around 300 novel families contain ~44% of over 5,000 unclassified mature miRNAs in miRBase 17, but it is still hard to say whether there are overlaps between these novel families and those existing small families, and what is the portion. Actually, finding the minority from the majority is still an open issue from the perspective of machine learning. Currently, one possible solution is to combine fast machine learning methods and traditional sequence alignment methods. First, constructing a family architecture only includes big families while keeping the others in a candidate pool. Then, performing sequence alignment for each miRNA in the pool to get its homologies and determine its family.

## Conclusions

Researchers working in both wet and dry labs can get benefit from miRNA family information [18,51]. But the traditional manual or semi-automatic miRNA classification methods can't deal well with the rapid growth of miRNAs, especially after the widespread of NGS technologies. The development of *miRFam *fills the gap. As the first miRNA-oriented family classification method, it validates that automatic method can be greatly helpful to solve the problem and using *n*-gram features is a satisfactory choice to describe miRNA primary sequences [37], which thus avoids the traditional multiple sequence alignment procedure.

In this work, we aim to deal with shorter maturities, which is more difficult to tackle compared with the longer precursors. Although we still use *n*-grams but this time a larger number of *n*-grams are considered. Intuitively, it will be much easier to correctly assign the miRNAs based on more sophisticated description schemes. Here, the question is also obvious, that the speed will be significantly affected. Furthermore, we introduce three different dimension reduction methods that have been validated to be effective in many fields. Finally, comparative studies show that Isomap is a better choice when selecting a feature subset to represent the whole *n*-gram feature set. Surprisingly, by reducing feature dimension, not only the speed of miRCluster is greatly enhanced, but also the performance is also slightly improved. Thus makes our method more efficient and effective.

A significant difference between miRCluster and *miRFam *is that miRCluster can recognize both novel members and novel families, while *miRFam *can only identify the former. Although miRCluster can only achieve comparable performances compared with *miRFam *in most situations, but considering that it does not require any prior knowledge, which makes it more practical and powerful in real use.

## Competing interests

The authors declare that they have no competing interests.

## Authors' contributions

LW and JD designed the methods and experiments. LW conducted the experiments and drafted the paper; JD analyzed the data and participated in writing the paper. T. Jin helped to prepare the data sets. JG and SZ supervised the research, and revised the paper.

## Supplementary Material

Additional file 1 **Supplemental materials for miRCluster.pdf**. All 3 additional tables and 2 additional figures are compiled into one file. These tables and figures could give more details of results showed in the main text and support conclusions we made in this article. Click here for file
