# Supplementary material for: Automatically clustering large-scale miRNA sequences: methods and experiments
Source: BMC Genomics. 2012 Dec 17;13(Suppl 8):S15. doi: 10.1186/1471-2164-13-S8-S15 (PMC3535721; doi:10.1186/1471-2164-13-S8-S15)
Supplement: Additional file 1 — Supplemental materials for miRCluster.pdf. All 3 additional tables and 2 additional figures are compiled into one file. These tables and figures could give more details of results showed in the main text and support conclusions we made in this article. [file 1471-2164-13-S8-S15-S1.pdf]

Additional file 1

Table S1- "Dead" families in miRBase16 before and after feature selection

	Family size	Family number	Family name list
Dead families before feature selection	18	1	mir-1422
	12	1	mir-6
	11	2	mir-466,mir-932
	10	1	mir-63
	9	2	MIR529,MIR818
	8	2	MIR1063,mir-4000
	7	8	MIR1023,MIR167_2,mir-1175,mir-2024,mir-465,mir-541 mir-769,mir-81
	6	8	mir-1296,mir-2147,mir-298,mir-668,mir-711,mir-744,mir-877,mir-957
	5	17	MIR1509,MIR774,mir-1273,mir-1274,mir-1282,mir-1388 ,mir-2808,mir-3065, mir-492,mir-676,mir-762,mir-84,mir-883,mir-92,mir-935, mir-980,mir-996
Dead families after features selection	14	1	mir-344
	12	2	MIR535,mir-337
	10	1	MIR1122
	9	1	mir-2733
	8	4	mir-1420,mir-325,mir-4000,mir-759
	7	10	MIR1507,MIR167_2,mir-1175,mir-1193,mir-2024,mir-465,mir-541,mir-632, mir-769,mir-891
	6	6	MIR2275,mir-1296,mir-1912,mir-2147,mir-298,mir-668
	5	15	MIR1222,MIR1509,MIR774,MIR824,mir-1179,mir-1207, mir-1273,mir-1274, mir-1388,mir-2808,mir-3065,mir-676,mir-84,mir-883,mir-92

Before feature selection, about 42 families with 285 mature sequences are not successfully discovered during the clustering stage. And after using Isomap to select 150 features, the dead families are reduced to 40 and mature sequences are 270.

Table S2- Detail of discovered new families in miRBase17

Family name	Family size	Discovered members before feature selection	Discovered members after feature selection
mir-3851	12	9	11
mir-3811	10	8	10
MIR5067	8	3	2
MIR3980	4	4	2
mir-2788	4	2	2
mir-3804	4	2	4
mir-3836	4	2	0
mir-4520	4	2	2
mir-4659	4	0	4
mir-3817	4	2	0

After selecting features with Isomap, the number of correctly clustered new families is decreased from 9 to 8. Two small families (mir-3836, mir-3817) are dead, but bigger families (mir-3851, mir-3811) are better clustered than before.

Table S3- Seed region weighting experiment on plant families

	**Top10**	**Top30**	**Families which has no less than 5 members**
No weighting	0.993224	0.974629	0.933432
Seed region weighted	0.992256	0.977104	0.936391

In plant, we try to treat the nucleotides in and out the seed region differently. The accuracy of before and after seed region weighting strategy are shown here.

Figure S1- Details of discovered novel families in miRBase17

This is an example of discovered novel families. The miRNA with a star in front of its name means it is unclassified in miRBase. (A) Features: Gram4, Cluster number: 800. (B) Features: Gram4, Cluster number: 1200. (C) Features: use Isomap to select 140 features from Gram4, Cluster number: 1200. (D) Features: use Isomap to select 140 features from Gram5, Cluster number: 1200.

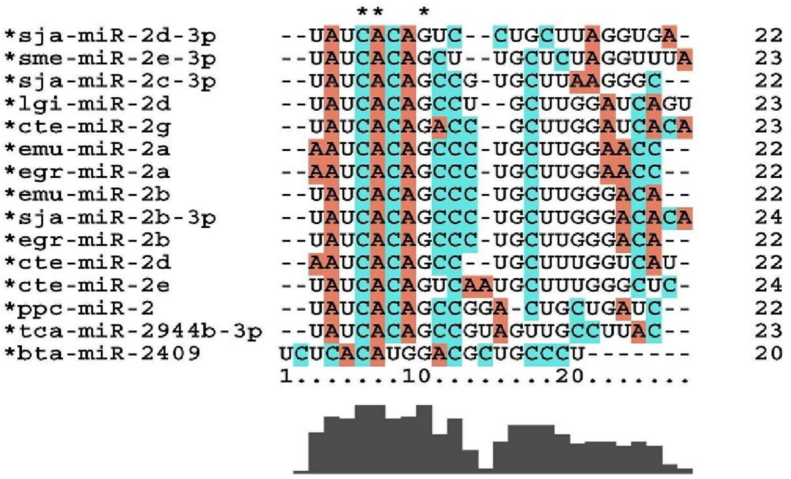

Cluster Number=800, Gram 4.

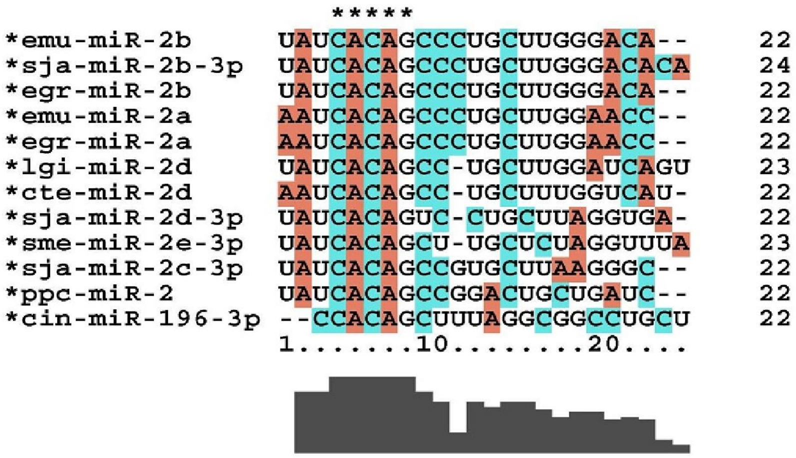

Cluster Number=1200, Gram 4.

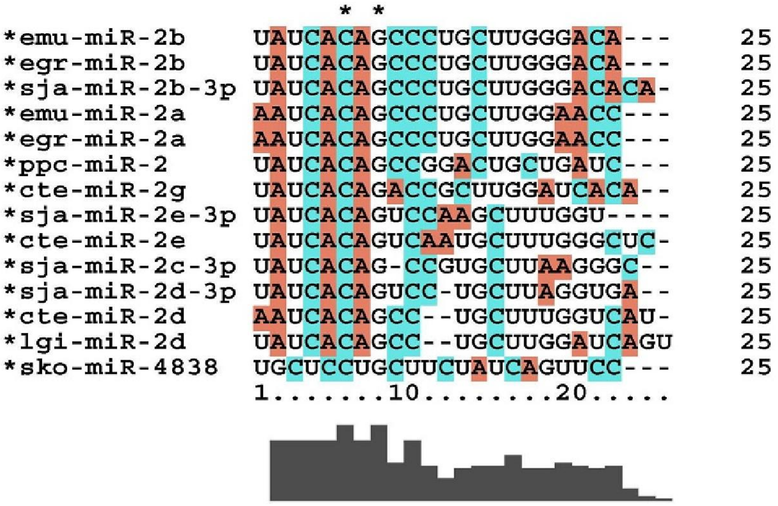

Cluster Number=1200, Gram 4, Isomap Dimension=140

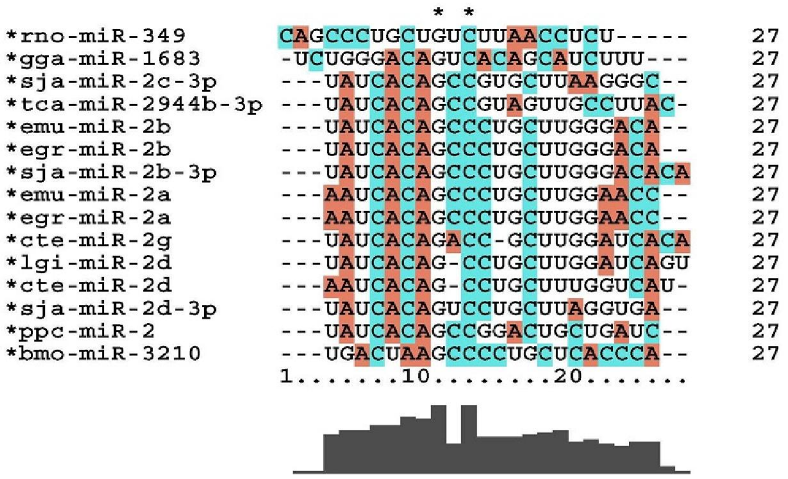

Cluster Number=1200, Gram 5, Isomap Dimension=140

Figure S2- Detail of discovered novel families mixed with known families in miRBase17

An example cluster of known families and novel miRNAs mixed together before and after feature selection. (A) Features: Gram4, Cluster number: 1200. (B) Features: 140 selected features by Isomap from Gram4. Cluster number: 1200. (C) Features: 140 selected features by Isomap from Gram5. Cluster number: 1200.

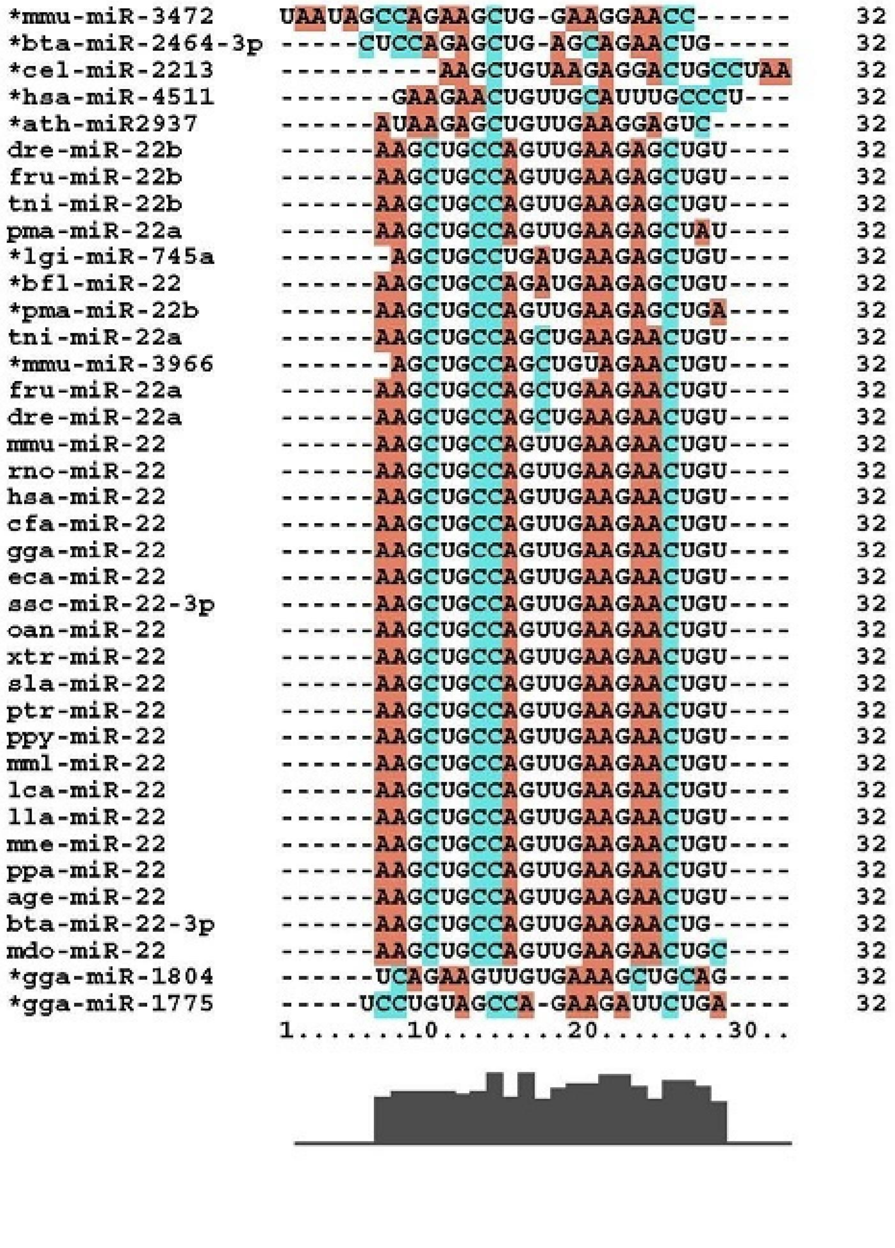

Cluster Number=1200, Gram 4.

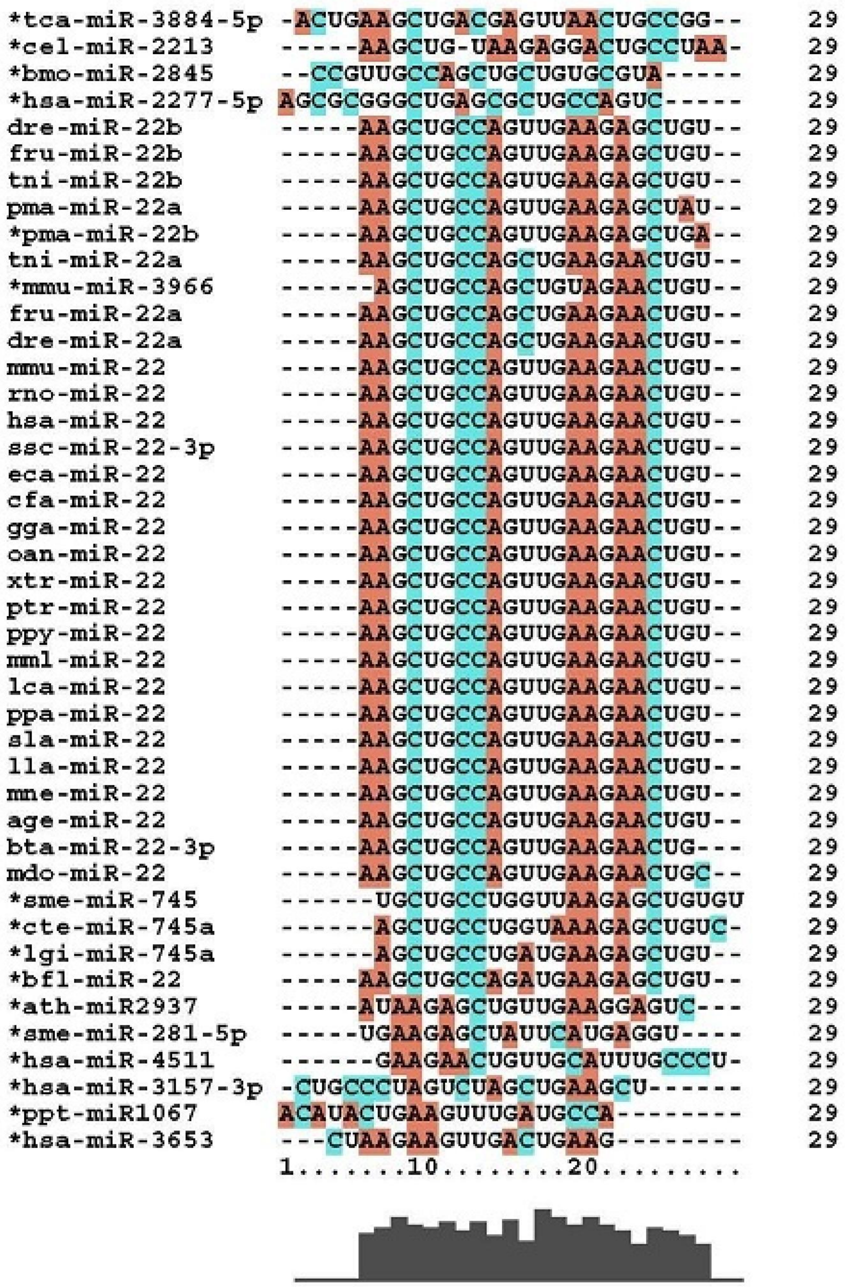

Cluster Number=1200, Gram 4, Isomap Dimension=140

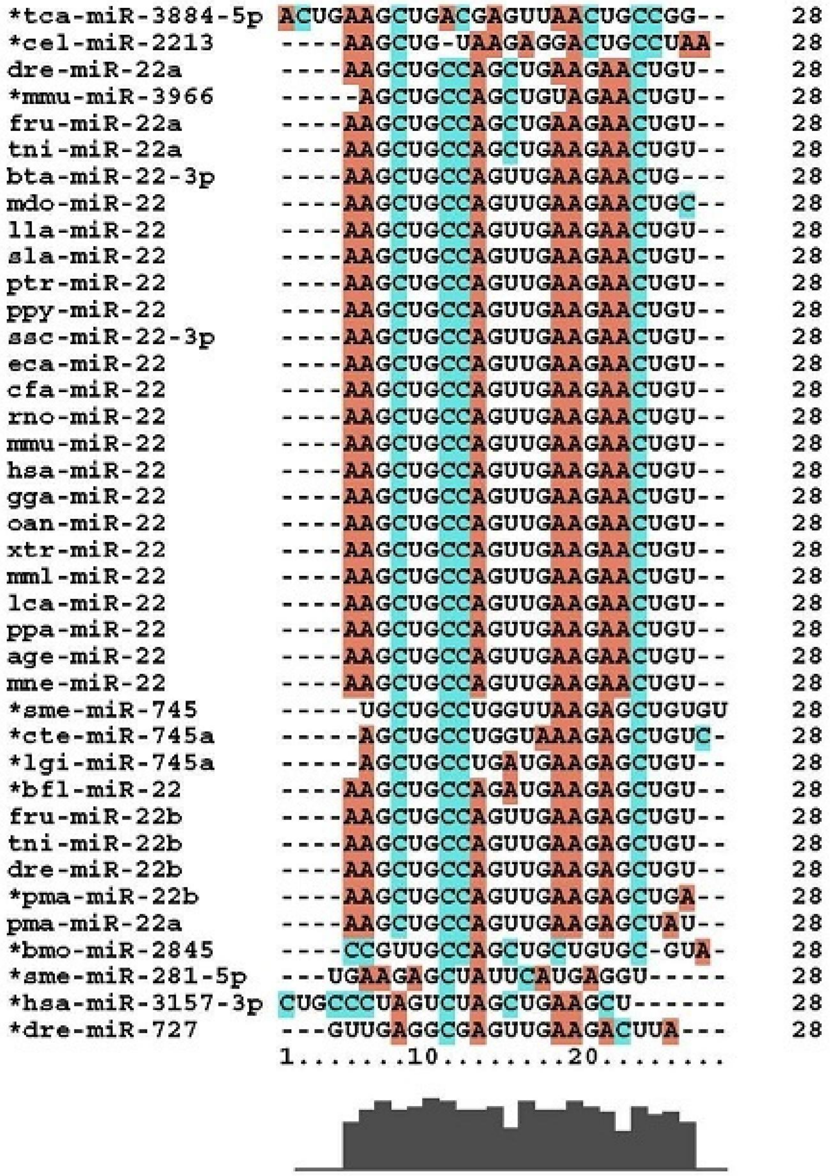

Cluster Number=1200, Gram 5, Isomap Dimension=140
